# Martingale inequalities for spline sequences

**DOI:** 10.1007/s11117-019-00668-2

**Published:** 2019-03-30

**Authors:** Markus Passenbrunner

**Affiliations:** Institute of Analysis, Johannes Kepler University Linz, Altenberger Strasse 69, 4040 Linz, Austria

**Keywords:** Martingale inequalities, Polynomial spline spaces, Orthogonal projection operators, 65D07, 60G42, 42C10

## Abstract

We show that D. Lépingle’s $L_1(\ell _2)$-inequality

$$\begin{aligned} \left\| \left( \sum _n {\mathbb {E}}[f_n | {\mathscr {F}}_{n-1}]^2 \right) ^{1/2}\right\| _1 \le 2\cdot \left\| \left( \sum _n f_n^2 \right) ^{1/2} \right\| _1, \quad f_n\in {\mathscr {F}}_n, \end{aligned}$$

extends to the case where we substitute the conditional expectation operators with orthogonal projection operators onto spline spaces and where we can allow that $f_n$ is contained in a suitable spline space ${\mathscr {S}}({\mathscr {F}}_n)$. This is done provided the filtration $({\mathscr {F}}_n)$ satisfies a certain regularity condition depending on the degree of smoothness of the functions contained in ${\mathscr {S}}({\mathscr {F}}_n)$. As a by-product, we also obtain a spline version of $H_1$-${{\,\mathrm{BMO}\,}}$ duality under this assumption.

## Introduction

This article is part of a series of papers that extend martingale results to polynomial spline sequences of arbitrary order (see e.g. [11, 14, 16–19, 22]). In order to explain those martingale type results, we have to introduce a little bit of terminology: Let *k* be a positive integer, $({\mathscr {F}}_n)$ an increasing sequence of $\sigma$-algebras of sets in [0, 1] where each ${\mathscr {F}}_n$ is generated by a finite partition of [0, 1] into intervals of positive length. Moreover, define the spline space

$$\begin{aligned} {\mathscr {S}}_k({\mathscr {F}}_n) = \{f\in C^{k-2}[0,1] : f\text { is a polynomial of order }k \text { on each atom of }{\mathscr {F}}_n \} \end{aligned}$$

and let $P_n^{(k)}$ be the orthogonal projection operator onto ${\mathscr {S}}_k({\mathscr {F}}_n)$ with respect to the $L_2$ inner product on [0, 1] with the Lebesgue measure $|\cdot |$. The space ${\mathscr {S}}_1({\mathscr {F}}_n)$ consists of piecewise constant functions and $P_n^{(1)}$ is the conditional expectation operator with respect to the $\sigma$-algebra ${\mathscr {F}}_n$. Similarly to the definition of martingales, we introduce the following notion: let $(f_n)_{n\ge 0}$ be a sequence of integrable functions. We call this sequence a *k-martingale spline sequence* (adapted to $({\mathscr {F}}_n)$) if, for all *n*,

$$\begin{aligned} P_n^{(k)} f_{n+1} = f_n. \end{aligned}$$

For basic facts about martingales and conditional expectations, we refer to [15].

Classical martingale theorems such as Doob’s inequality or the martingale convergence theorem in fact carry over to *k*-martingale spline sequences corresponding to *arbitrary* filtrations (${\mathscr {F}}_n$) of the above type, just by replacing conditional expectation operators by the projection operators $P_n^{(k)}$. Indeed, we have

(i) (Shadrin’s theorem) there exists a constant $C_k$ depending only on *k* such that
  $$\begin{aligned} \sup _n\Vert P_n^{(k)} : L_1 \rightarrow L_1 \Vert \le C_k, \end{aligned}$$
(ii) (Doob’s weak type inequality for splines)
  there exists a constant $C_k$ depending only on *k* such that for any *k*-martingale spline sequence $(f_n)$ and any $\lambda >0$,
  $$\begin{aligned} |\{ \sup _n |f_n| > \lambda \}| \le C_k \frac{\sup _n\Vert f_n\Vert _{1}}{ \lambda }, \end{aligned}$$
(iii) (Doob’s $L_p$ inequality for splines)
  for all $p\in (1,\infty ]$ there exists a constant $C_{p,k}$ depending only on *p* and *k* such that for all *k*-martingale spline sequences $(f_n)$,
  $$\begin{aligned} \big \Vert \sup _n |f_n| \big \Vert _{p} \le C_{p,k} \sup _n\Vert f_n\Vert _{p},\ \end{aligned}$$
(iv) (Spline convergence theorem)
  if $(f_n)$ is an $L_1$-bounded *k*-martingale spline sequence, then $(f_n)$ converges almost surely to some $L_1$-function,
(v) (Spline convergence theorem, $L_p$-version)
  for $1<p<\infty$, if $(f_n)$ is an $L_p$-bounded *k*-martingale spline sequence, then $(f_n)$ converges almost surely and in $L_p$.

Property (i) was proved by Shadrin in the groundbreaking paper [22]. We also refer to the paper [25] by von Golitschek, who gives a substantially shorter proof of (i). Properties (ii) and (iii) are proved in [19] and properties (iv) and (v) in [14], but see also [18], where it is shown that, in analogy to the martingale case, the validity of (iv) and (v) for all *k*-martingale spline sequences with values in a Banach space *X* characterize the Radon–Nikodým property of *X* (for background information on that material, we refer to the monographs [6, 20]).

Here, we continue this line of transferring martingale results to *k*-martingale spline sequences and extend  Lépingle’s $L_1(\ell _2)$-inequality [12], which reads

1.1 $$\begin{aligned} \Big \Vert \big ( \sum _n {\mathbb {E}}[f_n | \mathscr {F}_{n-1}]^2 \big )^{1/2}\Big \Vert _1 \le 2\cdot \Big \Vert \big ( \sum _n f_n^2 \big )^{1/2} \Big \Vert _1, \end{aligned}$$

provided the sequence of (real-valued) random variables $f_n$ is adapted to the filtration $({\mathscr {F}}_n)$, i.e. each $f_n$ is ${\mathscr {F}}_n$-measurable. Different proofs of (1.1) were given by Bourgain [3, Proposition 5], Delbaen and Schachermayer [4, Lemma 1] and Müller [13, Proposition 4.1]. The spline version of inequality (1.1) is contained in Theorem 4.1.

This inequality is an $L_1$ extension of the following result for $1<p<\infty$, proved by Stein [24], that holds for *arbitrary* integrable functions $f_n$:

1.2 $$\begin{aligned} \Big \Vert \big ( \sum _n {\mathbb {E}}[f_n |{\mathscr {F}}_{n-1}]^2 \big )^{1/2}\Big \Vert _p \le a_p \Big \Vert \big ( \sum _n f_n^2\big )^{1/2} \Big \Vert _p, \end{aligned}$$

for some constant $a_p$ depending only on *p*. This can be seen as a dual version of Doob’s inequality $\Vert \sup _{\ell } |{\mathbb {E}}[f | {\mathscr {F}}_\ell ]| \Vert _p \le c_p \Vert f\Vert _p$ for $p>1$, see [1]. Once we know Doob’s inequality for spline projections, which is point (iii) above, the same proof as in [1] works for spline projections if we use suitable positive operators $T_n$ instead of $P_n^{(k)}$ that also satisfy Doob’s inequality and dominate the operators $P_n^{(k)}$ pointwise (cf. Sects. 3.1, 3.2).

The usage of those operators $T_n$ is also necessary in the extension of inequality (1.1) to splines.  Lépingle’s proof of (1.1) rests on an idea by Herz [10] of splitting ${\mathbb {E}} [f_n \cdot h_n]$ (for $f_n$ being ${\mathscr {F}}_n$-measurable) by Cauchy–Schwarz after introducing the square function $S_n^2 = \sum _{\ell \le n} f_\ell ^2$:

1.3 $$\begin{aligned} ({\mathbb {E}}[f_n\cdot h_n])^2 \le {\mathbb {E}}[ f_n^2/S_n ] \cdot {\mathbb {E}}[S_n h_n^2] \end{aligned}$$

and estimating both factors on the right hand side separately. A key point in estimating the second factor is that $S_n$ is $\mathscr {F}_n$-measurable, and therefore, ${\mathbb {E}}[S_n|{\mathscr {F}}_n]=S_n$. If we want to allow $f_n\in {\mathscr {S}}_k({\mathscr {F}}_n)$, $S_n$ will not be contained in ${\mathscr {S}}_k({\mathscr {F}}_n)$ in general. Under certain conditions on the filtration $({\mathscr {F}}_n)$, we will show in this article how to substitute $S_n$ in estimate (1.3) by a function $g_n\in {\mathscr {S}}_k(\mathscr {F}_n)$ that enjoys similar properties to $S_n$ and allows us to proceed (cf. Sect. 3.4, in particular Proposition 3.4 and Theorem 3.6). As a by-product, we obtain a spline version (Theorem 4.2) of C. Fefferman’s theorem [7] on $H^1$-${{\,\mathrm{BMO}\,}}$ duality. For its martingale version, we refer to A. M. Garsia’s book [8] on Martingale Inequalities.

## Preliminaries

In this section, we collect all tools that are needed subsequently.

### Properties of polynomials

We will need Remez’ inequality for polynomials:

#### Theorem 2.1

Let $V\subset {\mathbb {R}}$ be a compact interval in ${\mathbb {R}}$ and $E\subset V$ a measurable subset. Then, for all polynomials *p* of order *k* (i.e. degree $k-1$) on *V*,

$$\begin{aligned} \Vert p \Vert _{L_\infty (V)} \le \bigg ( 4 \frac{|V|}{|E|}\bigg )^{k-1} \Vert p \Vert _{L_\infty (E)}. \end{aligned}$$

Applying this theorem with the set $E = \{x\in V : |p(x)| \le 8^{-k+1}\Vert p\Vert _{L_\infty (V)} \}$ immediately yields the following corollary:

#### Corollary 2.2

Let *p* be a polynomial of order *k* on a compact interval $V\subset {\mathbb {R}}$. Then

$$\begin{aligned} \big |\big \{ x \in V : |p(x)| \ge 8^{-k+1} \Vert p\Vert _{L_\infty (V)} \big \}\big | \ge |V|/2. \end{aligned}$$

### Properties of spline functions

For an interval $\sigma$-algebra ${\mathscr {F}}$ (i.e. ${\mathscr {F}}$ is generated by a finite collection of intervals having positive length), the space ${\mathscr {S}}_k({\mathscr {F}})$ is spanned by a very special local basis $(N_i)$, the so called B-spline basis. It has the properties that each $N_i$ is non-negative and each support of $N_i$ consists of at most *k* neighboring atoms of ${\mathscr {F}}$. Moreover, $(N_i)$ is a partition of unity, i.e. for all $x\in [0,1]$, there exist at most *k* functions $N_i$ so that $N_i(x)\ne 0$ and $\sum _i N_i(x)=1$. In the following, we denote by $E_i$ the support of the B-spline function $N_i$. The usual ordering of the B-splines $(N_i)$–which we also employ here–is such that for all *i*, $\inf E_i \le \inf E_{i+1}$ and $\sup E_i \le \sup E_{i+1}$.

We write $A(t)\lesssim B(t)$ to denote the existence of a constant *C* such that for all *t*, $A(t)\le C B(t)$, where *t* denote all implicit and explicit dependencies the expression *A* and *B* might have. If the constant *C* additionally depends on some parameter, we will indicate this in the text. Similarly, the symbols $\gtrsim$ and $\simeq$ are used.

Another important property of B-splines is the following relation between B-spline coefficients and the $L_p$-norm of the corresponding B-spline expansions.

#### Theorem 2.3

(B-spline stability, local and global) Let $1\le p\le \infty$ and $g=\sum _{j} a_j N_j$. Then, for all *j*,

2.1 $$\begin{aligned} |a_j|\lesssim |J_j|^{-1/p}\Vert g\Vert _{L_p(J_j)}, \end{aligned}$$

where $J_j$ is an atom of ${\mathscr {F}}$ contained in $E_j$ having maximal length. Additionally,

2.2 $$\begin{aligned} \Vert g\Vert _p\simeq \Vert (a_j|E_j|^{1/p})\Vert _{\ell _p}, \end{aligned}$$

where in both (2.1) and (2.2), the implied constants depend only on the spline order *k*.

Observe that (2.1) implies for $g\in \mathscr {S}_k({\mathscr {F}})$ and any measurable set $A\subset [0,1]$

2.3 $$\begin{aligned} \Vert g\Vert _{L_\infty (A)} \lesssim \max _{j : |E_j\cap A|>0} \Vert g\Vert _{L_\infty (J_j)}. \end{aligned}$$

We will also need the following relation between the B-spline expansion of a function and its expansion using B-splines of a finer grid.

#### Theorem 2.4

Let ${\mathscr {G}}\subset {\mathscr {F}}$ be two interval $\sigma$-algebras and denote by $(N_{{\mathscr {G}},i})_i$ the B-spline basis of the coarser space ${\mathscr {S}}_k({\mathscr {G}})$ and by $(N_{\mathscr {F},i})_i$ the B-spline basis of the finer space $\mathscr {S}_k({\mathscr {F}})$. Then, given $f=\sum _{j} a_j N_{{\mathscr {G}},j}$, we can expand *f* in the basis $(N_{{\mathscr {F}},i})_i$

$$\begin{aligned} \sum _j a_j N_{{\mathscr {G}},j} = \sum _i b_i N_{{\mathscr {F}},i}, \end{aligned}$$

where for each *i*, $b_i$ is a convex combination of the coefficients $a_j$ with ${\text {supp}}N_{{\mathscr {G}},j} \supseteq {\text {supp}}N_{{\mathscr {F}},i}$.

For those results and more information on spline functions, in particular B-splines, we refer to [21] or [5].

### Spline orthoprojectors

We now use the B-spline basis of ${\mathscr {S}}_k({\mathscr {F}})$ and expand the orthogonal projection operator *P* onto $\mathscr {S}_k({\mathscr {F}})$ in the form

2.4 $$\begin{aligned} Pf = \sum _{i,j} a_{ij} \Big (\int _0^1 f(x) N_i(x)\,\mathrm {d}x\Big )\cdot N_j \end{aligned}$$

for some coefficients $(a_{ij})$. Denoting by $E_{ij}$ the smallest interval containing both supports $E_i$ and $E_j$ of the B-spline functions $N_i$ and $N_j$ respectively, we have the following estimate for $a_{ij}$ [19]: there exist constants *C* and $0<q<1$ depending only on *k* so that for each interval $\sigma$-algebra ${\mathscr {F}}$ and each *i*, *j*,

2.5 $$\begin{aligned} |a_{ij}| \le C \frac{q^{|i-j|}}{|E_{ij}|}. \end{aligned}$$

### Spline square functions

Let $({\mathscr {F}}_n)$ be a sequence of increasing interval $\sigma$-algebras in [0, 1] and we assume that each $\mathscr {F}_{n+1}$ is generated from ${\mathscr {F}}_n$ by the subdivision of exactly one atom of ${\mathscr {F}}_n$ into two atoms of $\mathscr {F}_{n+1}$. Let $P_n$ be the orthogonal projection operator onto ${\mathscr {S}}_k({\mathscr {F}}_n)$. We denote $\Delta _n f = P_n f - P_{n-1} f$ and define the spline square function

$$\begin{aligned} Sf = \Big (\sum _n |\Delta _n f|^2\Big )^{1/2}. \end{aligned}$$

We have Burkholder’s inequality for the spline square function, i.e. for all $1<p<\infty$ [16], the $L_p$-norm of the square function *Sf* is comparable to the $L_p$-norm of *f*:

2.6 $$\begin{aligned} \Vert Sf\Vert _p \simeq \Vert f\Vert _p,\qquad f\in L_p \end{aligned}$$

with constants depending only on *p* and *k*. Moreover, for $p=1$, it is shown in [9] that

2.7 $$\begin{aligned} \Vert Sf\Vert _1 \simeq \sup _{\varepsilon \in \{-1,1\}^{{\mathbb {Z}}}} \Vert \sum _n \varepsilon _n \Delta _n f \Vert _1, \qquad Sf\in L_1, \end{aligned}$$

with constants depending only on *k* and where the proof of the $\lesssim$-part only uses Khintchine’s inequality whereas the proof of the $\gtrsim$-part uses fine properties of the functions $\Delta _n f$.

### $L_p(\ell _q)$-spaces

For $1\le p,q\le \infty$, we denote by $L_p(\ell _q)$ the space of sequences of measurable functions $(f_n)$ on [0, 1] so that the norm

$$\begin{aligned} \Vert (f_n)\Vert _{L_p(\ell _q)} = \Big (\int _0^1 \Big ( \sum _{n} |f_n(t)|^q \Big )^{p/q}\,\mathrm {d}t\Big )^{1/p} \end{aligned}$$

is finite (with the obvious modifications if $p=\infty$ or $q=\infty$). For $1\le p,q <\infty$, the dual space (see [2]) of $L_p(\ell _q)$ is $L_{p'}(\ell _{q'})$ with $p'=p/(p-1)$, $q'=q/(q-1)$ and the duality pairing

$$\begin{aligned} \langle (f_n), (g_n)\rangle = \int _0^1 \sum _n f_n(t) g_n(t) \,\mathrm {d}t. \end{aligned}$$

Hölder’s inequality takes the form $|\langle (f_n), (g_n)\rangle | \le \Vert (f_n)\Vert _{L_p(\ell _q)} \Vert (g_n)\Vert _{L_{p'}(\ell _{q'})}$.

## Main results

In this section, we prove our main results. Section 3.1 defines and gives properties of suitable positive operators that dominate our (non-positive) operators $P_n= P_n^{(k)}$ pointwise. In Sect. 3.2, we use those operators to give a spline version of Stein’s inequality (1.2). A useful property of conditional expectations is the tower property $\mathbb E_{{\mathscr {G}}} {\mathbb {E}}_{{\mathscr {F}}} f = {\mathbb {E}}_{{\mathscr {G}}} f$ for ${\mathscr {G}}\subset {\mathscr {F}}$. In this form, it extends to the operators $(P_n)$, but not to the operators *T* from Sect. 3.1. In Sect. 3.3 we prove a version of the tower property for those operators. Section 3.4 is devoted to establishing a duality estimate using a spline square function, which is the crucial ingredient in the proofs of the spline versions of both Lépingle’s inequality (1.1) and $H_1$-${{\,\mathrm{BMO}\,}}$ duality in Sect. 4.

### The positive operators *T*

As above, let ${\mathscr {F}}$ be an interval $\sigma$-algebra on [0, 1], $(N_i)$ the B-spline basis of ${\mathscr {S}}_k({\mathscr {F}})$, $E_i$ the support of $N_i$ and $E_{ij}$ the smallest interval containing both $E_i$ and $E_j$. Moreover, let *q* be a positive number smaller than 1. Then, we define the linear operator $T = T_{{\mathscr {F}}, q, k}$ by

$$\begin{aligned} Tf(x) := \sum _{i,j} \frac{q^{|i-j|}}{|E_{ij}|} \langle f, \mathbb {1}_{E_i}\rangle \mathbb {1}_{E_j}(x) = \int _0^1 K(x,t) f(t)\,\mathrm {d}t, \end{aligned}$$

where the kernel $K=K_{T}$ is given by

$$\begin{aligned} K(x,t) = \sum _{i,j} \frac{q^{|i-j|}}{|E_{ij}|}\mathbb {1}_{E_i}(t)\cdot \mathbb {1}_{E_j}(x). \end{aligned}$$

We observe that the operator *T* is selfadjoint (w.r.t the standard inner product on $L_2$) and

3.1 $$\begin{aligned} k\le K_x := \int _0^1 K(x,t) \,\mathrm {d}t\le \frac{2(k+1)}{1-q},\qquad x\in [0,1], \end{aligned}$$

which, in particular, implies the boundedness of the operator *T* on $L_1$ and $L_\infty$:

$$\begin{aligned} \Vert Tf\Vert _1 \le \frac{2(k+1)}{1-q} \Vert f\Vert _1,\qquad \Vert Tf\Vert _\infty \le \frac{2(k+1)}{1-q}\Vert f\Vert _\infty . \end{aligned}$$

Another very important property of *T* is that it is a positive operator, i.e. it maps non-negative functions to non-negative functions and that *T* satisfies Jensen’s inequality in the form

3.2 $$\begin{aligned} \varphi (Tf(x)) \le K_x^{-1} T\big (\varphi ( K_x \cdot f)\big )(x),\qquad f\in L_1, x\in [0,1], \end{aligned}$$

for convex functions $\varphi$. This is seen by applying the classical Jensen inequality to the probability measure $K(t,x)\,\mathrm {d}t/K_x$.

Let ${\mathscr {Mf}}f$ denote the Hardy–Littlewood maximal function of $f\in L_1$, i.e.

$$\begin{aligned} {\mathscr {M}} f(x) = \sup _{I\ni x} \frac{1}{|I|}\int _I |f(y)|\,\mathrm {d}y, \end{aligned}$$

where the supremum is taken over all subintervals of [0, 1] that contain the point *x*. This operator is of weak type (1, 1), i.e.

$$\begin{aligned} |\{ {\mathscr {M}} f> \lambda \}| \le C \lambda ^{-1} \Vert f\Vert _1, \qquad f\in L_1, \lambda >0 \end{aligned}$$

for some constant *C*. Since trivially we have the estimate $\Vert {\mathscr {M}}f\Vert _\infty \le \Vert f\Vert _\infty$, by Marcinkiewicz interpolation, for any $p>1$, there exists a constant $C_p$ depending only on *p* so that

$$\begin{aligned} \Vert {\mathscr {M}f}\Vert _p \le C_p \Vert f\Vert _p. \end{aligned}$$

For those assertions about ${\mathscr {M}}$, we refer to (for instance) [23].

The significance of *T* and ${\mathscr {M}}$ at this point is that we can use formula (2.4) and estimate (2.5) to obtain the pointwise bound

3.3 $$\begin{aligned} |Pf(x)| \le C_1 (T|f|)(x) \le C_2 {\mathscr {Mf}}(x),\qquad f\in L_1, x\in [0,1], \end{aligned}$$

where $T=T_{{\mathscr {F}},q,k}$ with *q* given by (2.5), $C_1$ is a constant that depends only on *k* and $C_2$ is a constant that depends only on *k* and the geometric progression *q*. But as the parameter $q<1$ in (2.5) depends only on *k*, the constant $C_2$ will also only depend on *k*.

In other words, (3.3) tells us that the positive operator *T* dominates the non-positive operator *P* pointwise, but at the same time, *T* is dominated by the Hardy–Littlewood maximal function ${\mathscr {M}}$ pointwise and independently of ${\mathscr {F}}$.

### Stein’s inequality for splines

We now use this pointwise dominating, positive operator *T* to prove Stein’s inequality for spline projections. For this, let $(\mathscr {F}_n)$ be an interval filtration on [0, 1] and $P_n$ be the orthogonal projection operator onto the space ${\mathscr {S}}_k(\mathscr {F}_n)$ of splines of order *k* corresponding to ${\mathscr {F}}_n$. Working with the positive operators $T_{{\mathscr {F}}_n, q, k}$ instead of the non-positive operators $P_n$, the proof of Stein’s inequality (1.2) for spline projections can be carried over from the martingale case (cf. [1, 24]). For completeness, we include it here.

#### Theorem 3.1

Suppose that $(f_n)$ is a sequence of arbitrary integrable functions on [0, 1]. Then, for $1\le r\le p<\infty$ or $1<p\le r\le \infty$,

3.4 $$\begin{aligned} \Vert (P_n f_n) \Vert _{L_p(\ell _r)} \lesssim \Vert (f_n) \Vert _{L_p(\ell _r)} \end{aligned}$$

where the implied constant depends only on *p*, *r* and *k*.

#### Proof

By (3.3), it suffices to prove this inequality for the operators $T_n=T_{{\mathscr {F}}_n,q,k}$ with *q* given by (2.5) instead of the operators $P_n$. First observe that for $r=p=1$, the assertion follows from Shadrin’s theorem ((i) on page 1). Inequality (3.3) and the $L_{p'}$-boundedness of ${\mathscr {M}}$ for $1<p'\le \infty$ imply that

3.5 $$\begin{aligned} \big \Vert \sup _{1\le n\le N} |T_n f| \big \Vert _{p'} \le C_{p',k} \Vert f \Vert _{p'}, \qquad f\in L_{p'} \end{aligned}$$

with a constant $C_{p',k}$ depending on $p'$ and *k*. Let $1\le p<\infty$ and $U_N : L_{p}(\ell _1^N) \rightarrow L_{p}$ be given by $(g_1,\ldots ,g_N)\mapsto \sum _{j=1}^N T_j g_j$. Inequality (3.5) implies the boundedness of the adjoint $U_N^* : L_{p'}\rightarrow L_{p'}(\ell _\infty ^N)$, $f\mapsto (T_j f)_{j=1}^N$ for $p'=p/(p-1)$ by a constant independent of *N* and therefore also the boundedness of $U_N$. Since $|T_j f| \le T_j|f|$ by the positivity of $T_j$, letting $N\rightarrow \infty$ implies (3.4) for $T_n$ instead of $P_n$ in the case $r=1$ and outer parameter $1\le p < \infty$.

If $1<r\le p$, we use Jensen’s inequality (3.2) and estimate (3.1) to obtain

$$\begin{aligned} \sum _{j=1}^N |T_j g_j|^r \lesssim \sum _{j=1}^N T_j(|g_j|^r) \end{aligned}$$

and apply the result for $r=1$ and the outer parameter *p* / *r* to get the result for $1\le r\le p<\infty$. The cases $1<p\le r\le \infty$ now just follow from this result using duality and the self-adjointness of $T_j$. $\square$

### Tower property of *T*

Next, we will prove a substitute of the tower property $$\mathbb E_{{\mathscr {G}}} {\mathbb {E}}_{{\mathscr {F}}} f={\mathbb {E}}_{{\mathscr {G}}}f$$$$({\mathscr {G}}\subset {\mathscr {F}})$$ for conditional expectations that applies to the operators *T*.

To formulate this result, we need a suitable notion of regularity for $\sigma$-algebras which we now describe. Let ${\mathscr {F}}$ be an interval $\sigma$-algebra, let $(N_j)$ be the B-spline basis of ${\mathscr {S}}_k({\mathscr {F}})$ and denote by $E_{j}$ the support of the function $N_j$. The *k-regularity parameter*$\gamma _k({\mathscr {F}})$ is defined as

$$\begin{aligned} \gamma _k({\mathscr {F}}) := \max _i \max ( |E_i| / |E_{i+1}|, |E_{i+1}| / |E_i| ), \end{aligned}$$

where the first maximum is taken over all *i* so that $E_i$ and $E_{i+1}$ are defined. The name *k*-regularity is motivated by the fact that each B-spline support $E_i$ of order *k* consists of at most *k* (neighboring) atoms of the $\sigma$-algebra ${\mathscr {F}}$.

#### Proposition 3.2

(Tower property of *T*) Let ${\mathscr {G}}\subset {\mathscr {F}}$ be two interval $\sigma$-algebras on [0, 1]. Let $S = T_{{\mathscr {G}},\sigma ,k}$ and $T=T_{\mathscr {F},\tau ,k'}$ for some $\sigma ,\tau \in (0,1)$ and some positive integers $k,k'$. Then, for all $q>\max (\tau ,\sigma )$, there exists a constant *C* depending on $q,k,k'$ so that

3.6 $$\begin{aligned} |ST f(x)| \le C \cdot \gamma ^k \cdot (T_{\mathscr {G},q,k} |f|)(x),\qquad f\in L_1, x\in [0,1], \end{aligned}$$

where $\gamma = \gamma _{k}({\mathscr {G}})$ denotes the *k*-regularity parameter of ${\mathscr {G}}$.

#### Proof

Let $(F_i)$ be the collection of B-spline supports in $\mathscr {S}_{k'}({\mathscr {F}})$ and $(G_i)$ the collection of B-spline supports in ${\mathscr {S}}_{k}({\mathscr {G}})$. Moreover, we denote by $F_{ij}$ the smallest interval containing $F_i$ and $F_j$ and by $G_{ij}$ the smallest interval containing $G_i$ and $G_j$.

We show (3.6) by showing the following inequality for the kernels $K_S$ of *S* and $K_T$ of *T* (cf. 3.1)

3.7 $$\begin{aligned} \int _0^1 K_{S}(x,t) K_{T}(t,s)\,\mathrm {d}t \le C \gamma ^k \sum _{i,j} \frac{q^{|i-j|}}{|G_{ij}|} \mathbb {1}_{G_i}(x) \mathbb {1}_{G_j}(s),\qquad x,s\in [0,1]\nonumber \\ \end{aligned}$$

for all $q>\max (\tau ,\sigma )$ and some constant *C* depending on $q,k,k'$. In order to prove this inequality, we first fix $x,s\in [0,1]$ and choose *i* such that $x\in G_i$ and $\ell$ such that $s\in F_\ell$. Moreover, based on $\ell$, we choose *j* so that $s\in G_j$ and $G_j \supset F_\ell$. There are at most $\max (k,k')$ choices for each of the indices $i,\ell ,j$ and without restriction, we treat those choices separately, i.e. we only have to estimate the expression

$$\begin{aligned} \sum _{m,r} \frac{\sigma ^{|m-i|} \tau ^{|r-\ell |} |G_m \cap F_r|}{|G_{im}| |F_{\ell r}|}. \end{aligned}$$

Since, for each *r*, there are also at most $k+k'-1$ indices *m* so that $|G_m\cap F_r| >0$ (recall that ${\mathscr {G}}\subset \mathscr {F}$), we choose one such index $m=m(r)$ and estimate

$$\begin{aligned} \Sigma = \sum _r \frac{\sigma ^{|m(r)-i|} \tau ^{|r-\ell |} |G_{m(r)} \cap F_r|}{|G_{i,m(r)}| |F_{\ell r}|}. \end{aligned}$$

Now, observe that for any parameter choice of *r* in the above sum,

$$\begin{aligned} G_{i,m(r)} \cup F_{\ell r} \supseteq (G_{ij}{\setminus } G_j) \cup G_i \end{aligned}$$

and therefore, since also $G_{m(r)}\cap F_r \subset G_{i,m(r)} \cap F_{\ell r}$,

$$\begin{aligned} \Sigma \le \frac{2}{|(G_{ij}{\setminus } G_j) \cup G_i|}\sum _r \sigma ^{|m(r) - i|}\tau ^{|r-\ell |}, \end{aligned}$$

which, using the *k*-regularity parameter $\gamma = \gamma _{k}({\mathscr {G}})$ of the $\sigma$-algebra ${\mathscr {G}}$ and denoting $\lambda = \max (\tau ,\sigma )$, we estimate by

$$\begin{aligned} \Sigma&\le \frac{2\gamma ^k}{|G_{ij}|} \sum _m \lambda ^{|m-i|}\sum _{r: m(r)=m} \lambda ^{|r-\ell |} \lesssim \frac{\gamma ^k}{|G_{ij}|}\sum _m \lambda ^{|i-m| + |m-j|} \\&\lesssim \frac{\gamma ^k}{|G_{ij}|} \big (|i-j|+1\big )\lambda ^{|i-j|}, \end{aligned}$$

where the implied constants depend on $\lambda ,k,k'$ and the estimate $\sum _{r: m(r)=m} \lambda ^{|r-\ell |} \lesssim \lambda ^{|m-j|}$ used the fact that, essentially, there are more atoms of ${\mathscr {F}}$ between $F_r$ and $F_\ell$ (for *r* as in the sum) than atoms of ${\mathscr {G}}$ between $G_m$ and $G_j$. Finally, we see that for any $q>\lambda$,

$$\begin{aligned} \Sigma \lesssim C\gamma ^k\frac{q^{|i-j|}}{|G_{ij}|} \end{aligned}$$

for some constant *C* depending on $q,k,k'$, and, as $x\in G_i$ and $s\in G_j$, this shows inequality (3.7). $\square$

As a corollary of Proposition 3.2, we have

#### Corollary 3.3

Let $(f_n)$ be functions in $L_1$. We denote by $P_n$ the orthogonal projection onto ${\mathscr {S}}_{k}({\mathscr {F}}_n)$ and by $P_n'$ the orthogonal projection onto ${\mathscr {S}}_{k'}({\mathscr {F}}_n)$ for some positive integers $k,k'$. Moreover, let $T_n$ be the operator $T_{{\mathscr {F}}_n, q, k}$ from (3.3) dominating $P_n$ pointwise.

Then, for any integer *n* and for any $1\le p\le \infty$,

$$\begin{aligned} \Big \Vert \sum _{\ell \ge n} P_n \big ((P_{\ell -1}' f_\ell )^2\big ) \Big \Vert _p \lesssim \Big \Vert \sum _{\ell \ge n} T_n \big ((P_{\ell -1}' f_\ell )^2\big ) \Big \Vert _p \lesssim \gamma _{k}({\mathscr {F}}_n)^k\cdot \Big \Vert \sum _{\ell \ge n} f_\ell ^2\Big \Vert _p, \end{aligned}$$

where the implied constants only depend on *k* and $k'$.

We remark that by Jensen’s inequality and the tower property, this is trivial for conditional expectations ${\mathbb {E}}(\cdot | \mathscr {F}_n)$ instead of the operators $P_n, T_n, P_{\ell -1}'$ even with an absolute constant on the right hand side.

#### Proof

We denote by $T_n$ the operator $T_{{\mathscr {F}}_n, q, k}$ and by $T_n'$ the operator $T_{{\mathscr {F}}_n, q', k'}$, where the parameters $q,q'<1$ are given by inequality (3.3) depending on *k* and $k'$ respectively. Setting $U_n := T_{{\mathscr {F}}_n, \max (q,q')^{1/2}, k}$, we perform the following chain of inequalities, where we use the positivity of $T_n$ and (3.3), Jensen’s inequality for $T_{\ell -1}'$, the tower property for $T_n T_{\ell -1}'$ and the $L_p$-boundedness of $U_n$, respectively:

$$\begin{aligned} \Big \Vert \sum _{\ell \ge n} T_n\big ( (P_{\ell -1}' f_\ell )^2\big )\Big \Vert _p&\lesssim \Big \Vert \sum _{\ell \ge n} T_n \big ( (T_{\ell -1}' |f_\ell |)^2\big )\Big \Vert _p \\&\lesssim \Big \Vert \sum _{\ell \ge n} T_n \big (T_{\ell -1}' f_\ell ^2\big )\Big \Vert _p \\&\le \Vert T_n(T_{n-1}' f_n^2) \Vert _p + \Big \Vert \sum _{\ell> n} T_n \big (T_{\ell -1}' f_\ell ^2\big )\Big \Vert _p \\&\lesssim \Vert f_n^2 \Vert _p + \gamma _{k}({\mathscr {F}}_n)^k \cdot \Big \Vert \sum _{\ell > n} U_n(f_\ell ^2)\Big \Vert _p \\&\lesssim \gamma _{k}({\mathscr {F}}_n)^k \cdot \Big \Vert \sum _{\ell \ge n} f_\ell ^2 \Big \Vert _p, \end{aligned}$$

where the implied constants only depend on *k* and $k'$. $\square$

### A duality estimate using a spline square function

In order to give the desired duality estimate contained in Theorem 3.6, we need the following construction of a function $g_n\in {\mathscr {S}}_k({\mathscr {F}}_n)$ based on a spline square function.

#### Proposition 3.4

Let $(f_n)$ be a sequence of functions with $f_n\in \mathscr {S}_k({\mathscr {F}}_n)$ for all *n* and set

$$\begin{aligned} X_n:=\sum _{\ell \le n} f_\ell ^2. \end{aligned}$$

Then, there exists a sequence of non-negative functions $g_n\in {\mathscr {S}}_{k}({\mathscr {F}}_n)$ so that for each *n*,

1. $$g_n \le g_{n+1}$$,
2. $$X_n^{1/2} \le g_n$$
3. ${\mathbb {E}} g_n \lesssim {\mathbb {E}} X_n^{1/2}$, where the implied constant depends on *k* and on $\sup _{m\le n}\gamma _{k}({\mathscr {F}}_m)$.

For the proof of this result, we need the following simple lemma.

#### Lemma 3.5

Let $c_1$ be a positive constant and let $(A_j)_{j=1}^N$ be a sequence of atoms in ${\mathscr {F}}_n$. Moreover, let $\ell : \{1,\ldots , N\} \rightarrow \{1,\ldots , n\}$ and, for each $j\in \{1,\ldots , N\}$, let $B_j$ be a subset of an atom $D_j$ of $\mathscr {F}_{\ell (j)}$ with

3.8 $$\begin{aligned} |B_j| \ge c_1 \sum _{\begin{array}{c} i:\ell (i)\ge \ell (j), \\ D_i\subseteq D_j \end{array}} |A_i |. \end{aligned}$$

Then, there exists a map $\varphi$ on $\{1,\ldots , N\}$ so that

1. $$|\varphi (j)| = c_1 |A_j|$$ for all *j*,
2. $$\varphi (j) \subseteq B_j$$ for all *j*,
3. $$\varphi (i) \cap \varphi (j) = \emptyset $$ for all $$i\ne j$$.

#### Proof

Without restriction, we assume that the sequence $(A_j)$ is enumerated such that $\ell (j+1) \le \ell (j)$ for all $1\le j\le N-1$. We first choose $\varphi (1)$ as an arbitrary (measurable) subset of $B_1$ with measure $c_1|A_1|$, which is possible by assumption (3.8). Next, we assume that for $1\le j\le j_0$, we have constructed $\varphi (j)$ with the properties

1. $$|\varphi (j)| = c_1|A_j|$$,
2. $$\varphi (j)\subseteq B_j$$,
3. $$\varphi (j) \cap \cup _{i<j} \varphi (i) = \emptyset $$.

Based on that, we now construct $\varphi (j_0 + 1)$. Define the index sets $\Gamma = \{ i : \ell (i) \ge \ell (j_0 +1), D_i \subseteq D_{j_0+1} \}$ and $\Lambda = \{ i : i\le j_0+1, D_i\subseteq D_{j_0+1} \}$. Since we assumed that $\ell$ is decreasing, we have $\Lambda \subseteq \Gamma$ and by the nestedness of the $\sigma$-algebras ${\mathscr {F}}_n$, we have for $i\le j_0+1$ that either $D_i\subset D_{j_0+1}$ or $|D_i \cap D_{j_0+1}|=0$. This implies

$$\begin{aligned} \Big | B_{j_0+1}{\setminus } \bigcup _{i\le j_0} \varphi (i) \Big |&= |B_{j_0 + 1}| - \Big | B_{j_0+1}\cap \bigcup _{i\le j_0} \varphi (i) \Big | \\&\ge c_1 \sum _{i\in \Gamma } |A_i| - \Big | D_{j_0+1} \cap \bigcup _{i\le j_0} \varphi (i) \Big | \\&\ge c_1 \sum _{i\in \Lambda } |A_i| - \Big | \bigcup _{i\in \Lambda {\setminus }\{j_0+1\}} \varphi (i) \Big | \\&\ge c_1\sum _{i\in \Lambda } |A_i| - \sum _{i\in \Lambda {\setminus } \{j_0+1\}} c_1 |A_i| = c_1 |A_{j_0+1}|. \end{aligned}$$

Therefore, we can choose $\varphi (j_0 +1) \subseteq B_{j_0+1}$ that is disjoint to $\varphi (i)$ for any $i\le j_0$ and $|\varphi (j_0+1)| = c_1 |A_{j_0+1}|$ which completes the proof. $\square$

#### Proof of Proposition 3.4

Fix *n* and let $(N_{n,j})$ be the B-spline basis of $\mathscr {S}_{k}({\mathscr {F}}_n)$. Moreover, for any *j*, set $E_{n,j} = {\text {supp}}N_{n,j}$ and $a_{n,j} := \max _{\ell \le n} \max _{r : E_{\ell ,r} \supset E_{n,j}}\Vert X_{\ell } \Vert _{L_\infty (E_{\ell ,r})}^{1/2}$ and we define $\ell (j)\le n$ and *r*(*j*) so that $E_{\ell (j),r(j)} \supseteq E_{n,j}$ and $a_{n,j} = \Vert X_{\ell (j)} \Vert _{L_\infty (E_{\ell (j),r(j)})}^{1/2}$. Set

$$\begin{aligned} g_n := \sum _j a_{n,j} N_{n,j} \in {\mathscr {S}}_{k}({\mathscr {F}}_n) \end{aligned}$$

and it will be proved subsequently that this $g_n$ has the desired properties.

Property (1): In order to show $g_n\le g_{n+1}$, we use Theorem 2.4 to write

$$\begin{aligned} g_n = \sum _j a_{n,j} N_{n,j} = \sum _j \beta _{n,j} N_{n+1,j}, \end{aligned}$$

where $\beta _{n,j}$ is a convex combination of those $a_{n,r}$ with $E_{n+1,j} \subseteq E_{n,r}$, and thus

$$\begin{aligned} g_n \le \sum _j \big (\max _{r: E_{n+1,j}\subseteq E_{n,r}} a_{n,r}\big ) N_{n+1,j}. \end{aligned}$$

By the very definition of $a_{n+1,j}$, we have

$$\begin{aligned} \max _{r: E_{n+1,j} \subseteq E_{n,r}} a_{n,r} \le a_{n+1,j}, \end{aligned}$$

and therefore, $g_n\le g_{n+1}$ pointwise, since the B-splines $(N_{n+1,j})_j$ are nonnegative functions.

Property (2):  Now we show that $X_n^{1/2}\le g_n$. Indeed, for any $x\in [0,1]$,

$$\begin{aligned} g_n(x) = \sum _j a_{n,j} N_{n,j}(x) \ge \min _{j : E_{n,j}\ni x} a_{n,j} \ge \min _{j: E_{n,j}\ni x} \Vert X_n\Vert _{L_\infty (E_{n,j})}^{1/2}\ge X_n(x)^{1/2}, \end{aligned}$$

since the collection of B-splines $(N_{n,j})_j$ forms a partition of unity.

Property (3):  Finally, we show ${\mathbb {E}} g_n \lesssim {\mathbb {E}} X_n^{1/2}$, where the implied constant depends only on *k* and on $\sup _{m\le n} \gamma _k({\mathscr {F}}_m)$. By B-spline stability (Theorem 2.3), we estimate the integral of $g_n$ by

3.9 $$\begin{aligned} {\mathbb {E}} g_n \lesssim \sum _j |E_{n,j}| \cdot \Vert X_{\ell (j)}\Vert _{L_\infty (E_{\ell (j),r(j)})}^{1/2}, \end{aligned}$$

where the implied constant only depends on *k*. In order to continue the estimate, we next show the inequality

3.10 $$\begin{aligned} \Vert X_{\ell } \Vert _{L_\infty (E_{\ell ,r})} \lesssim \max _{s : |E_{\ell ,r} \cap E_{\ell ,s}| >0} \Vert X_{\ell }\Vert _{L_\infty (J_{\ell ,s})}, \end{aligned}$$

where by $J_{\ell ,s}$ we denote an atom of ${\mathscr {F}}_\ell$ with $J_{\ell ,s} \subset E_{\ell ,s}$ of maximal length and the implied constant depends only on *k*. Indeed, we use Theorem 2.3 in the form of (2.3) to get ($f_m\in {\mathscr {S}}_k({\mathscr {F}}_\ell )$ for $m\le \ell$)

3.11 $$\begin{aligned} \Vert X_\ell \Vert _{L_\infty (E_{\ell ,r})}\le & {} \sum _{m\le \ell } \Vert f_m\Vert _{L_\infty (E_{\ell ,r})}^2 \nonumber \\\lesssim & {} \sum _{m\le \ell } \sum _{s : |E_{\ell ,s} \cap E_{\ell ,r}|>0} \Vert f_m\Vert _{L_\infty (J_{\ell ,s})}^2 = \sum _{s : |E_{\ell ,s}\cap E_{\ell ,r}|>0} \sum _{m\le \ell } \Vert f_m \Vert _{L_\infty (J_{\ell ,s})}^2.\nonumber \\ \end{aligned}$$

Now observe that for atoms *I* of ${\mathscr {F}}_\ell$, due to the equivalence of *p*-norms of polynomials (cf. Corollary 2.2),

$$\begin{aligned} \sum _{m\le \ell } \Vert f_m\Vert _{L_\infty (I)}^2 \lesssim \sum _{m\le \ell }\frac{1}{|I|} \int _I f_m^2 = \frac{1}{|I|} \int _I X_\ell \le \Vert X_\ell \Vert _{L_\infty (I)}, \end{aligned}$$

which means that, inserting this in estimate (3.11),

$$\begin{aligned} \Vert X_\ell \Vert _{L_\infty (E_{\ell ,r})} \lesssim \sum _{s : |E_{\ell ,s}\cap E_{\ell ,r}|>0} \Vert X_\ell \Vert _{L_\infty (J_{\ell ,s})}, \end{aligned}$$

and, since there are at most *k* indices *s* so that $|E_{\ell ,s} \cap E_{\ell ,r}|>0$, we have established (3.10).

We define $K_{\ell ,r}$ to be an interval $J_{\ell ,s}$ with $|E_{\ell ,r}\cap E_{\ell ,s}|>0$ so that

$$\begin{aligned} \max _{s : |E_{\ell ,r} \cap E_{\ell ,s}| >0} \Vert X_{\ell }\Vert _{L_\infty (J_{\ell ,s})} = \Vert X_{\ell }\Vert _{L_\infty (K_{\ell ,r})}. \end{aligned}$$

This means, after combining (3.9) with (3.10), we have

3.12 $$\begin{aligned} {\mathbb {E}} g_n \lesssim \sum _j|J_{n,j}|\cdot \Vert X_{\ell (j)}\Vert _{L_\infty (K_{\ell (j),r(j)})}^{1/2}. \end{aligned}$$

We now apply Lemma 3.5 with the function $\ell$ and the choices

$$\begin{aligned} A_j&= J_{n,j}, \qquad D_j = K_{\ell (j),r(j)}, \\ B_j&= \Big \{ t\in D_j : X_{\ell (j)}(t) \ge 8^{-2(k-1)} \Vert X_{\ell (j)} \Vert _{L_\infty (D_j)} \Big \}. \end{aligned}$$

In order to see Assumption (3.8) of Lemma 3.5, fix the index *j* and let *i* be such that $\ell (i)\ge \ell (j)$. By definition of $D_i = K_{\ell (i), r(i)}$, the smallest interval containing $J_{n,i}$ and $D_i$ contains at most $2k-1$ atoms of ${\mathscr {F}}_{\ell (i)}$ and, if $D_{i}\subset D_j$, the smallest interval containing $J_{n,i}$ and $D_j$ contains at most $2k-1$ atoms of ${\mathscr {F}}_{\ell (j)}$. This means that, in particular, $J_{n,i}$ is a subset of the union *V* of 4*k* atoms of ${\mathscr {F}}_{\ell (j)}$ with $D_j\subset V$. Since each atom of ${\mathscr {F}}_n$ occurs at most *k* times in the sequence $(A_j)$, there exists a constant $c_1$ depending on *k* and $\sup _{u\le \ell (j)} \gamma _k({\mathscr {F}}_u)\le \sup _{u\le n} \gamma _k(\mathscr {F}_u)$ so that

$$\begin{aligned} |D_j| \ge c_1 \sum _{\begin{array}{c} i:\ell (i)\ge \ell (j) \\ D_i\subset D_j \end{array}} |A_i|, \end{aligned}$$

which, since $|B_j|\ge |D_j|/2$ by Corollary 2.2, shows that the assumption of Lemma 3.5 holds true and we get a function $\varphi$ so that $|\varphi (j)| = c_1|J_{n,j}|/2$, $\varphi (j) \subset B_j$, $\varphi (i)\cap \varphi (j)=\emptyset$ for all *i*, *j*. Using these properties of $\varphi$, we continue the estimate in (3.12) and write

$$\begin{aligned} {\mathbb {E}} g_n&\lesssim \sum _j |J_{n,j}| \cdot \Vert X_{\ell (j)} \Vert _{L_\infty ( D_j)}^{1/2} \le 8^{k-1}\cdot \sum _{j} \frac{|J_{n,j}|}{|\varphi (j)|} \int _{\varphi (j)} X_{\ell (j)}^{1/2} \\&=\frac{2}{c_1} \cdot 8^{k-1} \cdot \sum _j \int _{\varphi (j)} X_{\ell (j)}^{1/2} \\&\lesssim \sum _j \int _{\varphi (j)} X_n^{1/2} \le {\mathbb {E}} X_n^{1/2}, \end{aligned}$$

with constants depending only on *k* and $\sup _{u\le n}\gamma _k({\mathscr {F}}_u)$. $\square$

Employing this construction of $g_n$, we now give the following duality estimate for spline projections (for the martingale case, see for instance [8]). The martingale version of this result is the essential estimate in the proof of both Lépingle’s inequality (1.1) and the $H^1$-${{\,\mathrm{BMO}\,}}$ duality.

#### Theorem 3.6

Let $({\mathscr {F}}_n)$ be such that $\gamma :=\sup _{n} \gamma _k({\mathscr {F}}_n) < \infty$ and $(f_n)_{n\ge 1}$ a sequence of functions with $f_n\in {\mathscr {S}}_k({\mathscr {F}}_n)$ for each *n*. Additionally, let $h_n\in L_1$ be arbitrary. Then, for any *N*,

$$\begin{aligned} \sum _{n\le N} {\mathbb {E}}[|f_n \cdot h_n|] \lesssim \sqrt{2}\cdot {\mathbb {E}}\Big [ \Big (\sum _{\ell \le N} f_\ell ^2\Big )^{1/2}\Big ] \cdot \sup _{n\le N} \Vert P_n\big ( \sum _{\ell =n}^N h_\ell ^2 \big )\Vert _\infty ^{1/2}, \end{aligned}$$

where the implied constant is the same constant that appears in (3) of Proposition 3.4 and therefore only depends on *k* and $\gamma$.

#### Proof

Let $X_n:= \sum _{\ell \le n} f_\ell ^2$ and $f_\ell \equiv 0$ for $\ell > N$ and $\ell \le 0$. By Proposition 3.4, we choose an increasing sequence $(g_n)$ of functions with $g_0=0$, $g_n\in {\mathscr {S}}_{k}({\mathscr {F}}_n)$ and the properties $X_n^{1/2}\le g_n$ and ${\mathbb {E}} g_n \lesssim {\mathbb {E}} X_n^{1/2}$ where the implied constant depends only on *k* and $\gamma$. Then, apply Cauchy–Schwarz inequality by introducing the factor $g_n^{1/2}$ to get

$$\begin{aligned} \sum _n{\mathbb {E}} [ |f_n \cdot h_n|]= & {} \sum _n {\mathbb {E}} \left[ \left| \frac{f_n}{g_n^{1/2}}\cdot g_n^{1/2} h_n\right| \right] \\\le & {} \left[ \sum _n {\mathbb {E}} [f_n^2/g_n] \right] ^{1/2} \cdot \left[ \sum _n {\mathbb {E}}[g_n h_n^2] \right] ^{1/2}. \end{aligned}$$

We estimate each of the factors on the right hand side separately and set

$$\begin{aligned} \Sigma _1 :=\sum _n {\mathbb {E}} [f_n^2/g_n], \qquad \Sigma _2:=\sum _n{\mathbb {E}}[g_n h_n^2]. \end{aligned}$$

The first factor is estimated by the pointwise inequality $X_n^{1/2}\le g_n$:

$$\begin{aligned} \Sigma _1 = {\mathbb {E}} \left[ \sum _n \frac{f_n^2}{g_n}\right]&\le {\mathbb {E}} \left[ \sum _n \frac{f_n^2}{X_n^{1/2}}\right] \\&= {\mathbb {E}} \left[ \sum _n \frac{X_n - X_{n-1}}{X_n^{1/2}} \right] \le 2{\mathbb {E}} \sum _n (X_n^{1/2} - X_{n-1}^{1/2}) = 2 {\mathbb {E}} X_N^{1/2}. \end{aligned}$$

We continue with $\Sigma _2$:

$$\begin{aligned} \Sigma _2&= {\mathbb {E}} \left[ \sum _{\ell =1}^N g_\ell h_\ell ^2 \right] = {\mathbb {E}} \left[ \sum _{\ell =1}^N \sum _{n=1}^\ell (g_n - g_{n-1}) h_\ell ^2 \right] \\&= {\mathbb {E}} \left[ \sum _{n=1}^N (g_n - g_{n-1}) \cdot \sum _{\ell =n}^N h_\ell ^2 \right] \\&= {\mathbb {E}} \left[ \sum _{n=1}^N P_n(g_n - g_{n-1}) \cdot \sum _{\ell =n}^N h_\ell ^2 \right] \\&={\mathbb {E}} \left[ \sum _{n=1}^N (g_n - g_{n-1}) \cdot P_n\left( \sum _{\ell =n}^N h_\ell ^2\right) \right] \\&\le {\mathbb {E}} \left[ \sum _{n=1}^N (g_n - g_{n-1})\right] \cdot \sup _{1\le n\le N} \left\| P_n \left( \sum _{\ell =n}^N h_\ell ^2 \right) \right\| _\infty , \end{aligned}$$

where the last inequality follows from $g_n\ge g_{n-1}$. Noting that by the properties of $g_n$, ${\mathbb {E}} \big [\sum _{n=1}^N (g_n - g_{n-1})\big ] = {\mathbb {E}} g_N \lesssim {\mathbb {E}} X_N^{1/2}$ and combining the two parts $\Sigma _1$ and $\Sigma _2$, we obtain the conclusion. $\square$

## Applications

We give two applications of Theorem 3.6, (i) D. Lépingle’s inequality and (ii) an analogue of C. Fefferman’s $H_1$-${{\,\mathrm{BMO}\,}}$ duality in the setting of splines. Once the results from Sect. 3 are known, the proofs of the subsequent results proceed similarly to their martingale counterparts in [8, 12] by using spline properties instead of martingale properties.

### Lépingle’s inequality for splines

#### Theorem 4.1

Let $k,k'$ be positive integers. Let $({\mathscr {F}}_n)$ be an interval filtration with $\sup _n \gamma _k({\mathscr {F}}_n)<\infty$ and, for any *n*, $f_n \in {\mathscr {S}}_{k}({\mathscr {F}}_n)$ and $P_n'$ be the orthogonal projection operator on ${\mathscr {S}}_{k'}({\mathscr {F}}_n)$. Then,

$$\begin{aligned} \Vert (P_{n-1}' f_n) \Vert _{L_1(\ell _2)} = \left\| \left( \sum _n (P_{n-1}' f_n)^2 \right) ^{1/2} \right\| _1 \lesssim \left\| \left( \sum _n f_n^2\right) ^{1/2} \right\| _1 = \Vert (f_n) \Vert _{L_1(\ell _2)}, \end{aligned}$$

where the implied constant depends only on *k*, $k'$ and $\sup _n \gamma _{k}({\mathscr {F}}_n)$.

We emphasize that the parameters *k* and $k'$ can be different here, *k* being the spline order of the sequence $(f_n)$ and $k'$ being the spline order of the projection operators $P_{n-1}'$. In particular, the constant on the right hand side does not depend on the $k'$-regularity parameter $\sup _n \gamma _{k'}({\mathcal {F}}_n)$.

#### Proof

We first assume that $f_n=0$ for $n>N$ and begin by using duality

$$\begin{aligned} {\mathbb {E}} \left[ \left( \sum _n (P_{n-1}'f_n)^2\right) ^{1/2}\right] = \sup _{(H_n)} {\mathbb {E}} \left[ \sum _n (P_{n-1}' f_n) \cdot H_n\right] , \end{aligned}$$

where sup is taken over all $(H_n)\in L_\infty (\ell _2)$ with $\Vert (H_n)\Vert _{L_\infty (\ell _2)} =1$. By the self-adjointness of $P_{n-1}'$,

$$\begin{aligned} {\mathbb {E}}\big [(P_{n-1}'f_n) \cdot H_n\big ] = {\mathbb {E}}\big [ f_n \cdot (P_{n-1}' H_n) \big ]. \end{aligned}$$

Then we apply Theorem 3.6 for $f_n$ and $h_n=P_{n-1}'H_n$ to obtain (denoting by $P_n$ the orthogonal projection operator onto ${\mathscr {S}}_k({\mathscr {F}}_n)$)

4.1 $$\begin{aligned} \sum _{n\le N} | {\mathbb {E}} [f_n\cdot h_n] | \lesssim {\mathbb {E}}\left[ \left( \sum _{\ell \le N} f_\ell ^2\right) ^{1/2}\right] \cdot \sup _{n\le N} \left\| P_n \left( \sum _{\ell =n}^N (P_{\ell -1}'H_\ell )^2\right) \right\| _\infty ^{1/2}. \end{aligned}$$

To estimate the right hand side, we note that for any *n*, by Corollary 3.3,

$$\begin{aligned} \left\| P_n \left( \sum _{\ell =n}^N (P_{\ell -1}' H_\ell )^2\right) \right\| _\infty \lesssim \left\| \sum _{\ell =n}^N H_\ell ^2 \right\| _\infty . \end{aligned}$$

Therefore, (4.1) implies

$$\begin{aligned} {\mathbb {E}} \left[ \left( \sum _n (P_{n-1}'f_n)^2\right) ^{1/2}\right] = \sup _{(H_n)} {\mathbb {E}} \left[ \sum _n f_n \cdot ( P_{n-1}' H_n)\right] \lesssim {\mathbb {E}}\left[ \left( \sum _{\ell \le N} f_\ell ^2\right) ^{1/2}\right] , \end{aligned}$$

with a constant depending only on *k*,$k'$ and $\sup _{n\le N} \gamma _k({\mathscr {F}}_n)$. Letting *N* tend to infinity, we obtain the conclusion. $\square$

### $H_1$-${{\,\mathrm{BMO}\,}}$ duality for splines

We fix an interval filtration $({\mathscr {F}}_n)_{n=1}^\infty$, a spline order *k* and the orthogonal projection operators $P_n$ onto ${\mathscr {S}}_k({\mathscr {F}}_n)$ and additionally, we set $P_0=0$. For $f\in L_1$, we introduce the notation

$$\begin{aligned} \Delta _n f := P_n f - P_{n-1} f,\qquad S_n(f) := \left( \sum _{\ell =1}^n (\Delta _\ell f)^2\right) ^{1/2}, \qquad S(f) = \sup _n S_n(f). \end{aligned}$$

Observe that for $\ell < n$ and $f,g\in L_1$,

4.2 $$\begin{aligned} {\mathbb {E}} [ \Delta _\ell f \cdot \Delta _n g ] = {\mathbb {E}} [ P_\ell (\Delta _\ell f) \cdot \Delta _n g] = {\mathbb {E}} [ \Delta _\ell f \cdot P_\ell (\Delta _n g)] = 0. \end{aligned}$$

Let *V* be the $L_1$-closure of $\cup _n {\mathscr {S}}_k({\mathscr {F}}_n)$. Then, the uniform boundedness of $P_n$ on $L_1$ implies that $P_n f\rightarrow f$ in $L_1$ for $f\in V$. Next, set

$$\begin{aligned} H_{1,k} = H_{1,k}( ({\mathscr {F}}_n) ) = \{ f\in V : {\mathbb {E}} ( S(f) ) < \infty \} \end{aligned}$$

and equip $H_{1,k}$ with the norm $\Vert f \Vert _{H_{1,k}} = {\mathbb {E}} S(f)$. Define

$$\begin{aligned} {{\,\mathrm{BMO}\,}}_k = {{\,\mathrm{BMO}\,}}_k ( ({\mathscr {F}}_n) ) = \left\{ f\in V : \sup _n \Vert \sum _{\ell \ge n}T_n\big ( (\Delta _\ell f)^2 \big )\Vert _\infty < \infty \right\} \end{aligned}$$

and the corresponding quasinorm

$$\begin{aligned} \Vert f \Vert _{{{\,\mathrm{BMO}\,}}_k} = \sup _n \big \Vert \sum _{\ell \ge n} T_n\big ((\Delta _\ell f)^2 \big )\big \Vert _\infty ^{1/2}, \end{aligned}$$

where $T_n$ is the operator from (3.3) that dominates $P_n$ pointwise.

Let us now assume $\sup _n \gamma _k({\mathscr {F}}_n) < \infty$. In this case we identify, similarly to $H_1$-${{\,\mathrm{BMO}\,}}$-duality (cf. [7, 8, 10]), ${{\,\mathrm{BMO}\,}}_k$ as the dual space of $H_{1,k}$.

First, we use the duality estimate Theorem 3.6 and (4.2) to prove, for $f\in H_{1,k}$ and $h\in {{\,\mathrm{BMO}\,}}_k$,

$$\begin{aligned} \big | {\mathbb {E}} \big [ (P_n f) \cdot (P_n h) \big ] \big | \le \sum _{\ell \le n} {\mathbb {E}} \big [ |\Delta _\ell f| \cdot |\Delta _\ell h|\big ] \lesssim \Vert S_n(f) \Vert _1 \cdot \Vert h\Vert _{{{\,\mathrm{BMO}\,}}_k}. \end{aligned}$$

This estimate also implies that the limit $\lim _n {\mathbb {E}} \big [ (P_nf)\cdot (P_nh) \big ]$ exists and satisfies

$$\begin{aligned} \big |\lim _n {\mathbb {E}} \big [ (P_nf)\cdot (P_nh) \big ]\big | \lesssim \Vert f\Vert _{H_{1,k}} \cdot \Vert h\Vert _{{{\,\mathrm{BMO}\,}}_k}. \end{aligned}$$

On the other hand, similarly to the martingale case (see [8]), given a continuous linear functional *L* on $H_{1,k}$, we extend *L* norm-preservingly to a continuous linear functional $\Lambda$ on $L_1(\ell _2)$, which, by Sect. 2.5, has the form

$$\begin{aligned} \Lambda (\eta ) = {\mathbb {E}} \left[ \sum _\ell \sigma _\ell \eta _\ell \right] , \qquad \eta \in L_1(\ell _2) \end{aligned}$$

for some $\sigma \in L_\infty (\ell _2)$. The *k*-martingale spline sequence $h_n= \sum _{\ell \le n} \Delta _\ell \sigma _\ell$ is bounded in $L_2$ and therefore, by the spline convergence theorem ((v) on page 2), has a limit $h\in L_2$ with $P_n h = h_n$ and which is also contained in ${{\,\mathrm{BMO}\,}}_k$. Indeed, by using Corollary 3.3, we obtain $\Vert h\Vert _{{{\,\mathrm{BMO}\,}}_k} \lesssim \Vert \sigma \Vert _{L_\infty (\ell _2)} = \Vert \Lambda \Vert = \Vert L\Vert$ with a constant depending only on *k* and $\sup _{n}\gamma _k({\mathscr {F}}_n)$. Moreover, for $f\in H_{1,k}$, since *L* is continuous on $H_{1,k}$,

$$\begin{aligned} L(f) =\lim _n L(P_n f)&= \lim _n \Lambda \big ((\Delta _1 f,\ldots , \Delta _n f,0,0,\ldots ) \big ) \\&=\lim _n \sum _{\ell =1}^n {\mathbb {E}}[ \sigma _\ell \cdot \Delta _\ell f ]= \lim _n {\mathbb {E}}\big [ (P_nf)\cdot (P_nh) \big ]. \end{aligned}$$

This yields the following

#### Theorem 4.2

If $\sup _n \gamma _k({\mathscr {F}}_n)<\infty$, the linear mapping

$$\begin{aligned} u : {{\,\mathrm{BMO}\,}}_k \rightarrow H_{1,k}^*, \qquad h \mapsto \big ( f \mapsto \lim _n {\mathbb {E}}\big [(P_n f)\cdot (P_n h)\big ] \big ) \end{aligned}$$

is bijective and satisfies

$$\begin{aligned} \Vert u(h) \Vert _{H_{1,k}^*} \simeq \Vert h\Vert _{{{\,\mathrm{BMO}\,}}_k}, \end{aligned}$$

where the implied constants depend only on *k* and $\sup _n \gamma _k({\mathscr {F}}_n)$.

#### Remark 4.3

We close with a few remarks concerning the above result and we assume that $({\mathscr {F}}_n)$ is a sequence of increasing interval $\sigma$-algebras with $\sup _n \gamma _k({\mathscr {F}}_n) < \infty$.

1. By Khintchine’s inequality, $\Vert Sf\Vert _1 \lesssim \sup _{\varepsilon \in \{-1,1\}^{{\mathbb {Z}}}} \Vert \sum _n\varepsilon _n\Delta _n f\Vert _1$. Based on the interval filtration $({\mathscr {F}}_n)$, we can generate an interval filtration $({\mathscr {G}}_n)$ that contains $({\mathscr {F}}_n)$ as a subsequence and each ${\mathscr {G}}_{n+1}$ is generated from ${\mathscr {G}}_n$ by dividing exactly one atom of ${\mathscr {G}}_n$ into two atoms of $\mathscr {G}_{n+1}$. Denoting by $P_n^{{\mathscr {G}}}$ the orthogonal projection operator onto ${\mathscr {S}}_k({\mathscr {G}}_n)$ and $\Delta _j^{\mathscr {G}}= P_j^{{\mathscr {G}}}-P_{j-1}^{{\mathscr {G}}}$, we can write
  $$\begin{aligned} \sum _n\varepsilon _n\Delta _n f = \sum _n \varepsilon _n \sum _{j=a_n}^{a_{n+1}-1} \Delta _j^{{\mathscr {G}}} f \end{aligned}$$
  for some sequence $(a_n)$. By using inequalities (2.7) and (2.6) and writing $(S^{{\mathscr {G}}}f)^2= \sum _j |\Delta _j^{{\mathscr {G}}} f|^2$, we obtain for $p>1$
  $$\begin{aligned} \Vert Sf\Vert _1 \lesssim \Vert S^{{\mathscr {G}}} f\Vert _1 \le \Vert S^{{\mathscr {G}}} f\Vert _p \lesssim \Vert f\Vert _p. \end{aligned}$$
  This implies $L_p\subset H_{1,k}$ for all $p>1$ and, by duality, ${{\,\mathrm{BMO}\,}}_k \subset L_p$ for all $p<\infty$.
2. If $({\mathscr {F}}_n)$ is of the form that each $\mathscr {F}_{n+1}$ is generated from ${\mathscr {F}}_n$ by splitting exactly one atom of ${\mathscr {F}}_n$ into two atoms of ${\mathscr {F}}_{n+1}$ and under the condition $\sup _n \gamma _{k-1}({\mathscr {F}}_n) < \infty$ (which is stronger than $\sup _n \gamma _k({\mathscr {F}}_n)<\infty$), it is shown in [9] that
  $$\begin{aligned} \Vert Sf\Vert _{1} \simeq \Vert f\Vert _{H_1}, \end{aligned}$$
  where $H_1$ denotes the atomic Hardy space on [0, 1], i.e. in this case, $H_{1,k}$ coincides with $H_1$.
